# Indigenous neonatal facemask

**DOI:** 10.4103/0019-5049.65356

**Published:** 2010

**Authors:** Lalit Kumar Raiger

**Affiliations:** Department of Anaesthesiology, RNT Medical College, Udaipur, Rajasthan, India

Sir,

This report is to highlight how an emergency situation let to the creation of an indigenous mask to overcome an emergency situation. Routinely, the Jackson–Rees modification of Ayre’s T-piece breathing circuit with a Rendell Baker Soucek mask[1,2] is preferred for ventilation in neonatal anaesthesia, as this mask has a malleable edge to fit on the face with low dead space.[2]

Following an unfortunate electric short-circuit in one of the paediatric emergency OT, we had to shift the paediatric surgical emergencies to another OT. At midnight, a 3-day-old neonate requiring emergency laparotomy was posted for surgery and as the preparation for anaesthetic induction took place, the neonatal face mask was found missing.

This piece of equipment seems to have been left inadvertently in the disabled OT. Since there was no replacement at that time of the night we created our own indigenous neonatal face mask from empty disposable PVC-made fluid bottle.

**Figure 1:**
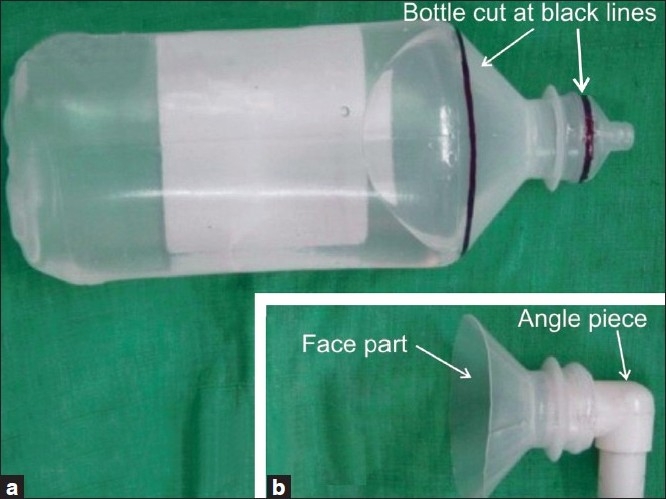
(a) Two black markings from where the bottle was cut; (b) newly formed face mask attached with the angle piece

The upper part of the bottle (tapering part) was cut transversely [Figure 1a], keeping the diameter of the bottle such that it comfortably covered the nose and mouth of the baby. Now the cap of the bottle, through which the intravenous drip-set spike is inserted, was cut to fit the patient end of Jackson–Ree’s circuit with an angle piece (15 mm) [Figure 1b].

Cotton and an adhesive tape were fixed to the circumference of the ‘face mask’ to prevent injury to the face. We were thus able to ventilate the neonate and proceed with anaesthesia uneventfully.
